# Sarcomatoid Squamous Cell Carcinoma in a 15-Year-Old Girl: A Report of a Rare Case

**DOI:** 10.7759/cureus.65767

**Published:** 2024-07-30

**Authors:** Prachi Surolia, Rajanikanth Kambala, Nitin Bhola, Anchal Agarwal

**Affiliations:** 1 Oral and Maxillofacial Surgery, Sharad Pawar Dental College and Hospital, Datta Meghe Institute of Higher Education and Research, Wardha, IND

**Keywords:** ca tongue, oral cancers, young females, squamous cell carcinoma of tongue, sarcomatoid variant

## Abstract

Sarcomatoid is a rare variant of squamous cell carcinoma. We present here the case of a 15-year-old female patient; she presented with an extra-oral fungation on the right side of her face and restricted tongue movements, diagnosed as sarcomatoid squamous cell carcinoma of the tongue on incisional biopsy. A positron emission tomography scan was advised on the first visit to rule out distant metastasis. Due to the previous history of no improvement after medical oncology management and the extent of the lesion, which made the disease inoperable, the patient was planned for the best supportive care.

## Introduction

Sarcomatoid carcinoma is an exceptionally rare and aggressive tumor that can develop in any organ, frequently in the head and neck, lungs, and genital tract of females [1]. Within the head and neck region, it is most commonly found in the larynx, secondly the oral cavity, and then the oropharynx; it can manifest in any head and neck region [2]. Despite the neck and head being a common site, its occurrence in the oral cavity is rare, comprising less than 1% of all oral cavity tumors. When it does appear in the oral cavity, it typically affects the alveolar ridge or gingiva (19%), tongue (20%), and lower lip (42%) [3].

The age at diagnosis ranges from 47 to 88 years, with an average age of 65.7 years, and the condition shows a higher prevalence in males [4]. The sarcomatoid variant of squamous cell carcinoma is notably more aggressive, with local recurrence rates between 52% and 73% and distant metastasis occurring in approximately 33% of cases [2,5].

## Case presentation

**Figure 1:**
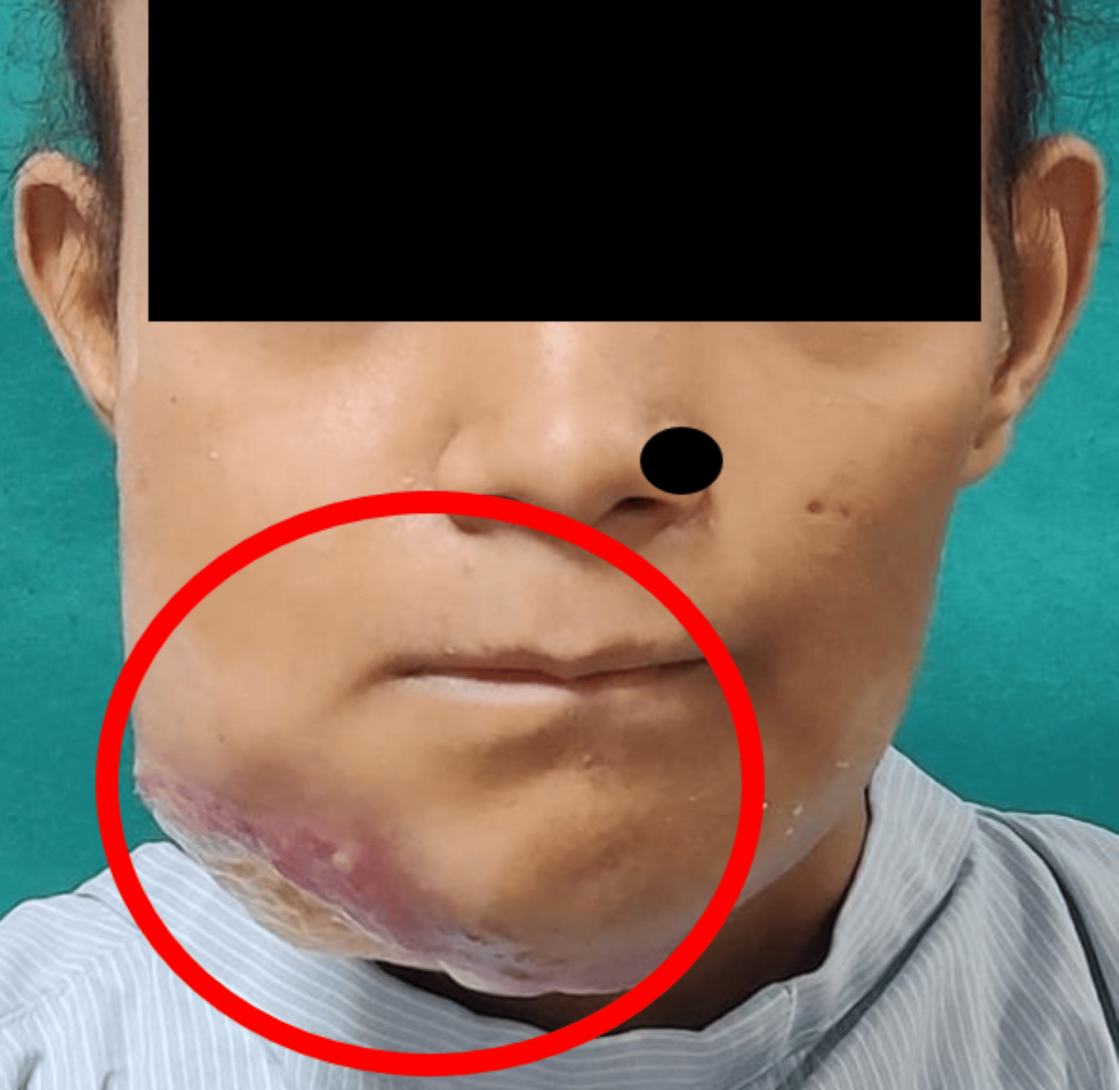
Frontal picture of the patient showing edema with extraoral fungation present on the right side of the face

A female patient aged 15 years came with a chief complaint of a non-healing ulcer over the right side of the tongue with restricted movements for 10 months. It was almost 10 months back when she noticed a non-healing ulcer, which was initially small in size and had increased to a size of approximately 10 x 11 cm intraorally. The patient gave a history of weight loss of 10 kg since 10 months, loss of appetite, and change in the consistency of saliva from thin to thick ropy. The patient denied having any deleterious habits. Extraorally, fungation with edema could be seen on the right side of the face (Figure 1).

**Figure 2:**
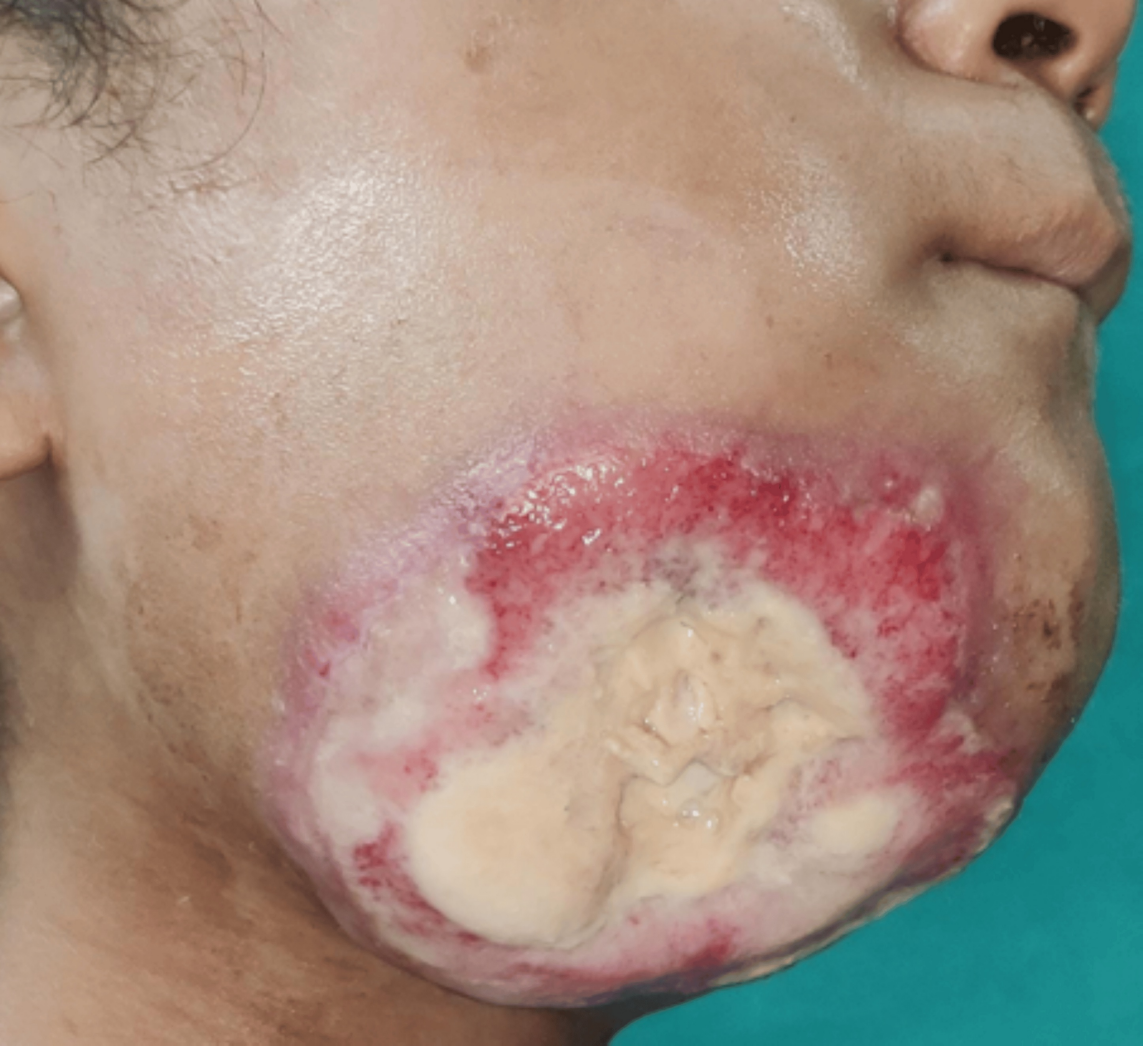
Extraoral fungation extended supero-inferiorly from the level of ala tragus line to 2 cm below the lower border of the mandible.

On extraoral examination, there was reduced mouth opening with restriction of jaw movements. The patient's face was grossly asymmetrical, the extraoral size was 7 x 6 cm approximately, the shape was roughly oval, the color was pinkish red, the consistency was hard, tenderness was present, and the local temperature was not raised. It extended anteriorly from the midline of the face to posteriorly 1 cm short of the right angle of the mandible, supero-inferiorly from the level of the right ala tragus line to 2 cm below the lower border of the mandible (Figure 2).

**Figure 3:**
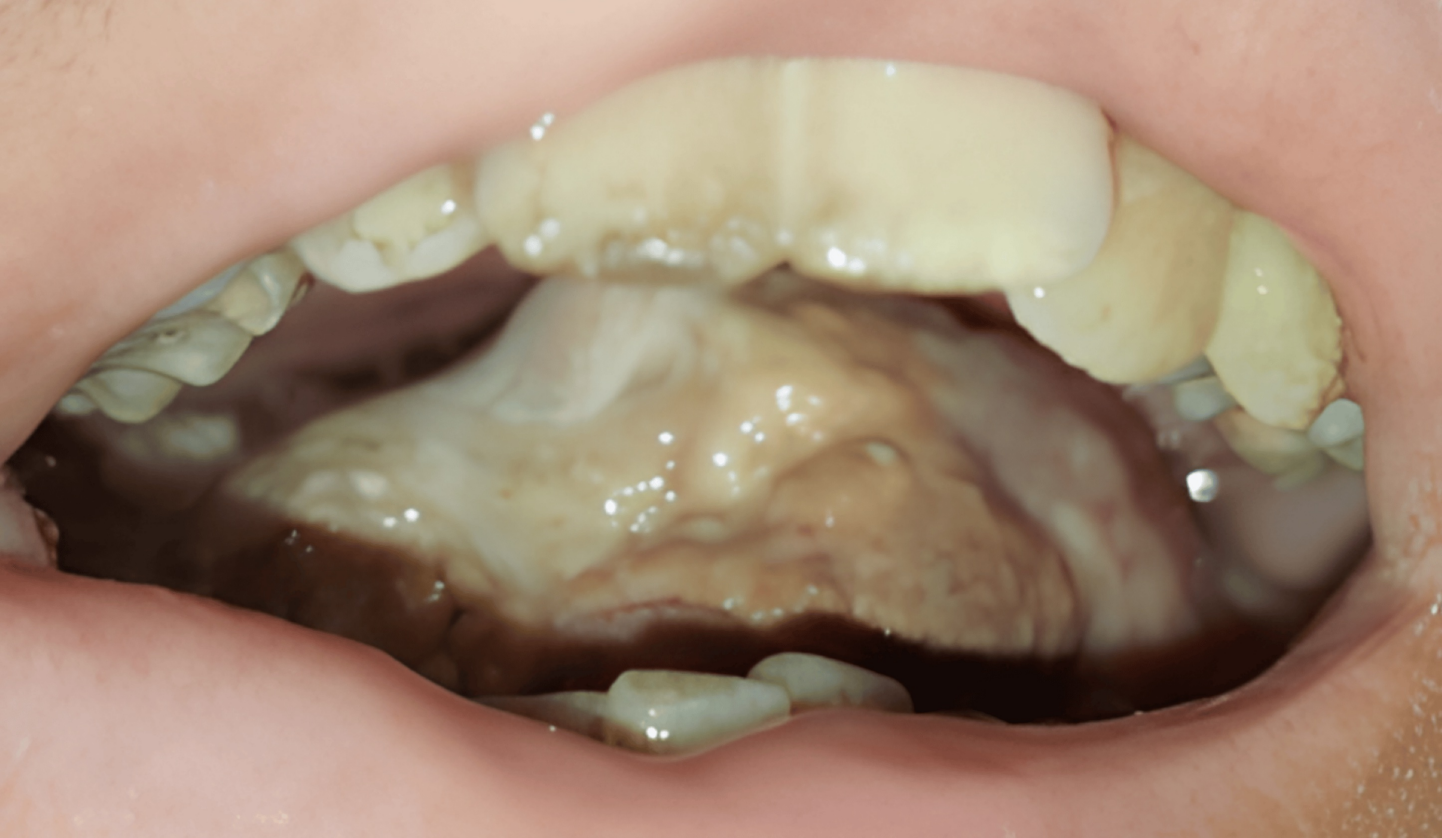
Lesion seen on the right lateral border of the tongue crossing the midline extending supero-inferiorly from the dorsal surface to the ventral surface of the tongue with restricted mouth opening.

On intraoral examination, a lesion was seen over the right lateral border crossing the midline of the tongue; supero-inferiorly, the lesion extended from the dorsal surface of the tongue to the ventral surface of the tongue and was hard in consistency, and there was restriction of tongue movements (Figure 3).

**Figure 4:**
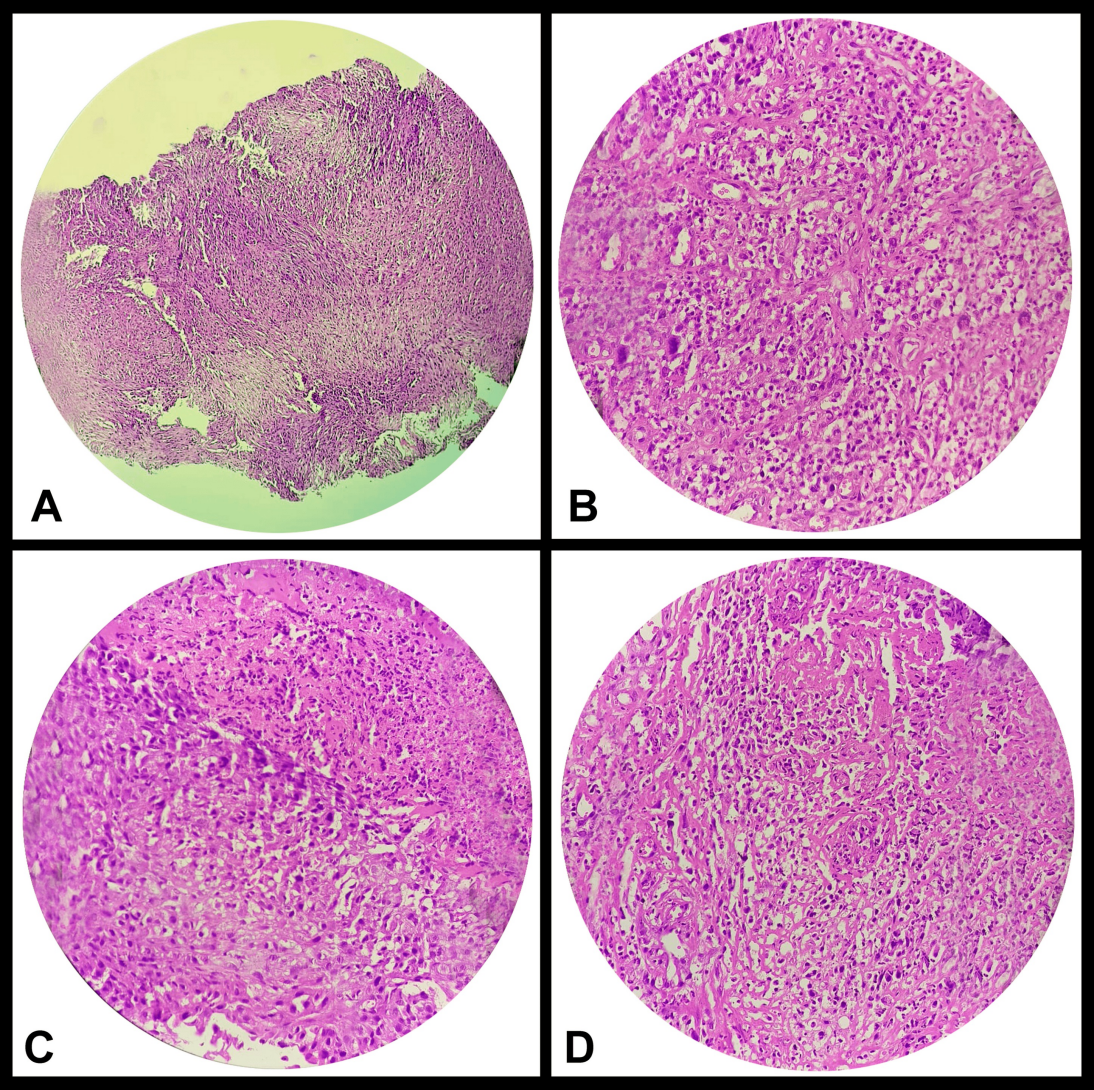
Histopathology showing sarcomatoid squamous cell carcinoma

The patient initially sought treatment at an external facility, where the biopsy was done, and the report was suggestive of sarcomatoid squamous cell carcinoma (Figure 4).

**Figure 5:**
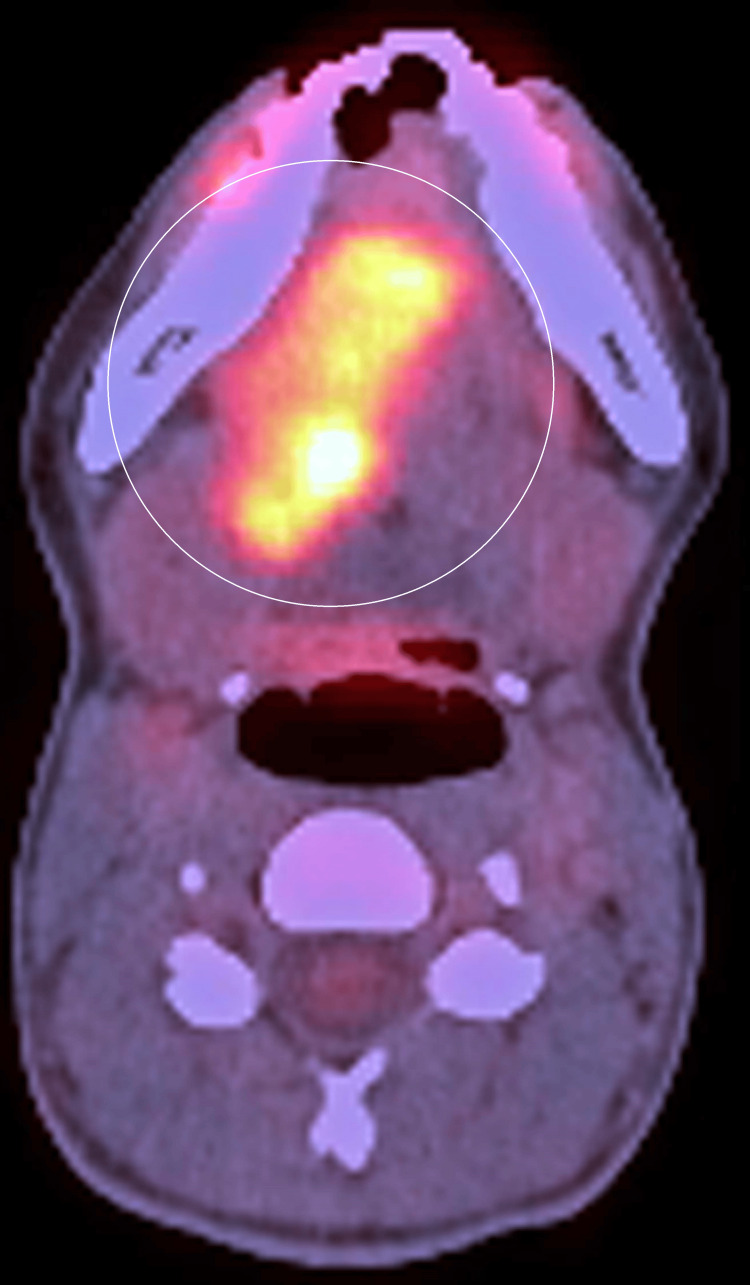
Hypermetabolic ulcerative proliferative lesion involving the entire right side of the tongue

The patient was then advised to undergo a PET CT scan. The results indicated a hypermetabolic ulcerative proliferative lesion involving the entire right side of the tongue (Figure 5).

**Figure 6:**
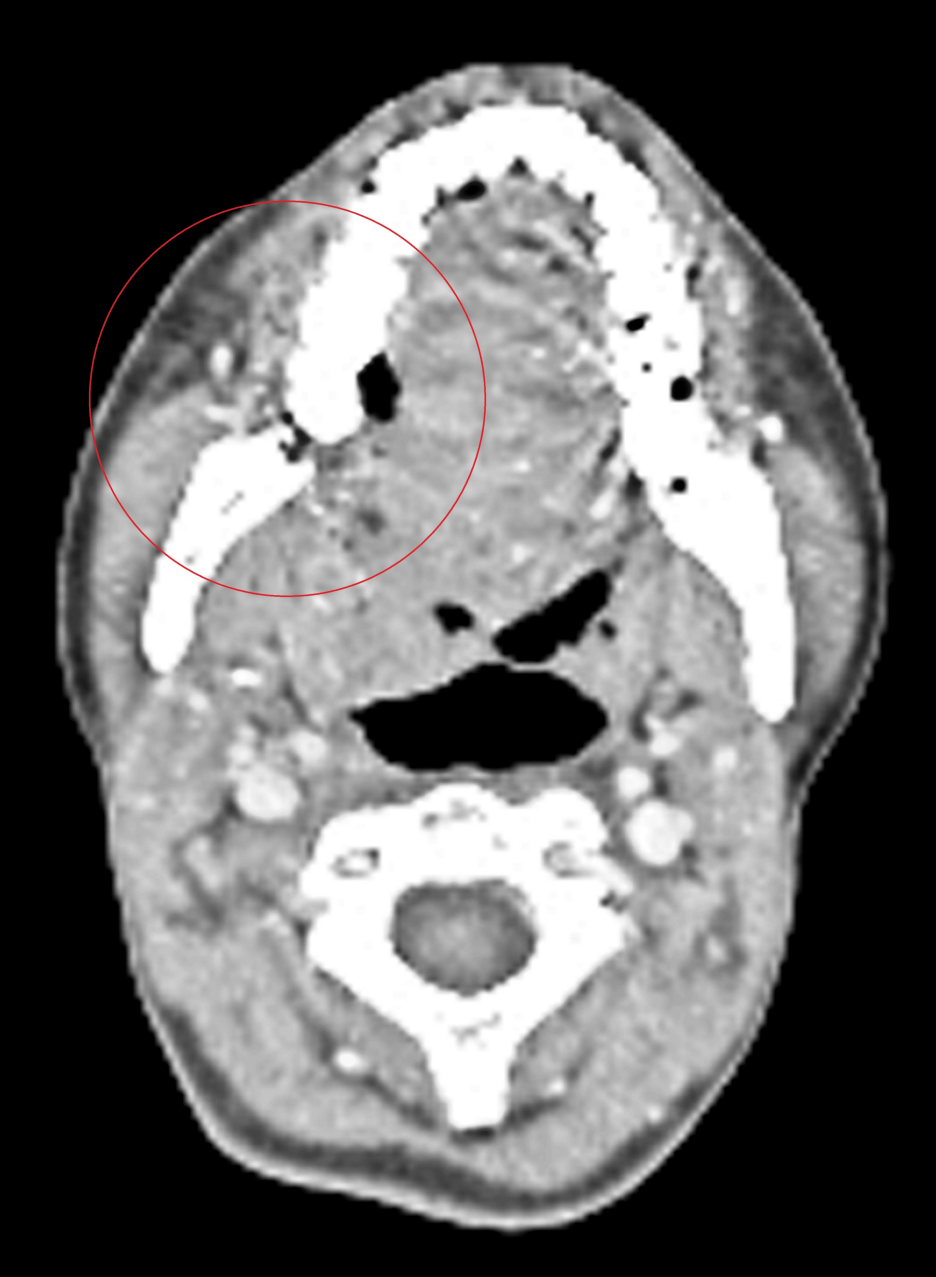
The lesion extended to the lip, lateral border, and crossed the midline to the base of the tongue, right tonsil, tonsillolingual sulcus, and vallecula

The lesion extended to the lip lateral border and crossed the midline to the base of the tongue, right tonsil, tonsillolingual sulcus, and vallecula, and extended inferiorly to the right side of the floor of the mouth (Figure 6).

The lesion measured 5.0 x 4.5 x 4.2 cm. Additionally, a metabolically active level Ib/submandibular gland node, the largest measuring 2 x 1.4 cm, showed metastasis. The patient was then advised for 20 cycles of curative neoadjuvant chemotherapy but received only four cycles of regimen, which was paclitaxel 80 mg, cisplatin 30 mg, and 5-fluorouracil 500 mg from February 2024 to March 2024. However, because of no improvement, the patient was referred to our center. Due to inoperability, the patient was planned for best supportive care.

## Discussion

Sarcomatoid carcinoma is a rare type of squamous cell carcinoma, and it is identified by the epithelial part consisting of poorly differentiated squamous cell carcinoma and proliferating spindle cell components in the mesenchymal part [1]. Sarcomatoid squamous cell carcinoma usually appears as large polypoid masses. Histologically, they are biphasic tumors that have been identified as monoclonal, dedifferentiated forms of conventional squamous cell carcinoma [6].

Markers

Immunohistochemical (IHC) markers vimentin and AE1 or AE3 are particularly useful in confirming oral sarcomatoid squamous cell carcinoma. A diagnostic algorithm for distinguishing the sarcomatoid variant of carcinomas from another neoplasm of spindle cells has been developed based on the analysis of 171 cases of mucosal spindle cell tumors in the head and neck. Sarcomatoid carcinoma typically shows positive immunoreactivity for cytokeratin and vimentin and negative immunoreactivity for S100 and CD34 [6].

Prognosis

Due to the increased tendency of the tumors to spread along planes of tissue and lymphatic drainage system, which leads to lymphatic and distant metastases, tumor exhibits a high mortality rate [1]. At the very initial period of diagnosis, the patient may have distant micrometastases [7]. During the disease progression, lymph node metastasis and distant metastasis to the brain, lungs, and other soft tissues may occur [6]. As compared to conventional squamous cell carcinoma, sarcomatoid carcinoma showed a higher mortality rate with a five-year survival rate of less than 16% in a study of 78 patients with tumors of the head and neck [1]. Several factors significantly contribute to a poor prognosis, like distant metastatic recurrence, advanced tumor stage (III/IV vs. I/II), vascular invasion, nodal disease, and advanced T classification (T3-4 vs. T1-2) [2]. A comprehensive PET CT scan can be valuable for detecting early distant metastasis in these patients. Additionally, adjuvant therapies such as radiotherapy, chemotherapy, and/or immunotherapy should be used in combination for optimal treatment [1].

Differential diagnosis

Malignant and benign tumors such as fibromatosis, reactive epithelial proliferations, squamous cell carcinoma, nodular fasciitis, fibrosarcoma, malignant peripheral nerve sheath tumor, malignant fibrous histiocytoma, leiomyosarcoma, rhabdomyosarcoma, malignant melanoma, and mesenchymal chondrosarcoma [8].

Management

This carcinoma typically is shown at an advanced stage, but clinicians can achieve a decent disease-free survival with the treatment of curative intent [9]. Wide local surgical excision is the preferred surgical method. While most experts agree with the idea of radiation therapy being generally ineffective, it is considered a viable option for patients who are not operable. Additionally, adjuvant radiation therapy may be helpful in cases with surgical margins positive or patients with metastasis at the node at diagnosis [10]. Previous studies have identified that the translocation of anaplastic lymphoma kinase can act as a significant oncogenic cause in sarcomatoid variants, and the advantages of the drug crizotinib have been demonstrated in cases with anaplastic lymphoma kinase translocation [11]. Therefore, knowledge of the genetic and molecular aspects is crucial for developing newer treatment options.

## Conclusions

Thus, a sarcomatoid variant of squamous cell carcinoma at a young age is rare. Due to the high tendency of this tumor to spread along planes of tissue and lymphatic drainage system, leading to early lymphatic and distant metastases, early identification and early treatment of the tumor is favorable. The mortality is higher with this type of variant, so treating the tumor at an early stage with curative intent may result in a favorable disease-free survival period.
